# A cluster investigation of *Candida auris* among hospitalized incarcerated patients

**DOI:** 10.1017/ash.2023.520

**Published:** 2023-12-19

**Authors:** April N. McDougal, Mary Ann DeMaet, Bobbiejean Garcia, Teresa York, Thomas Iverson, Olugbenga Ojo, Janak Patel

**Affiliations:** 1 Department of Infection Control and Healthcare Epidemiology, University of Texas Medical Branch, Galveston, TX, USA; 2 Division of Infectious Disease, Department of Internal Medicine, University of Texas Medical Branch, Galveston, TX, USA; 3 Healthcare Safety Unit, Texas Department of State Health Services, Austin, TX, USA; 4 Utah Department of Health and Human Services, Salt Lake City, UT, USA; 5 Division of General Internal Medicine, Department of Internal Medicine, University of Texas Medical Branch, Galveston, TX, USA; 6 Texas Department of Criminal Justice Hospital & Clinics, Galveston, TX, USA; 7 Division of Infectious Disease, Department of Pediatrics, University of Texas Medical Branch, Galveston, TX, USA

## Abstract

**Objective:**

Investigate and mitigate a cluster of *Candida auris* cases among incarcerated patients in a maximum-security prison hospital utilizing contact tracing, screening, whole genome sequencing, and environmental sampling and decontamination.

**Design:**

Outbreak investigation.

**Setting:**

Inpatient prison hospital affiliated with an academic tertiary referral center.

**Patients:**

Inmates of the Texas Department of Criminal Justice.

**Methods:**

Epidemiologic and environmental investigations were conducted including contact tracing, point prevalence surveys, and environmental sampling. Whole genome sequencing was performed on positive patient isolates.

**Results:**

Following a clinical case of *C. auris* fungemia, 344 patients underwent *C. auris* surveillance screening. Eight (2.3%) patients were identified with *C. auris* colonization. All patients were male. Our index patient was the only clinical case and death. Whole genome sequencing was performed on the nine patient isolates. All isolates were clade III (Africa) and clustered together with the largest SNP difference being 21. Environmental cultures from 7 of 61 rooms (11.5%) were positive following terminal disinfection with bleach. Sites nearest to the patient were most often positive including the hospital bed rails and bedside table. The transmission cluster was successfully mitigated within 60 days of identification.

**Conclusions:**

Implementation of an aggressive surveillance and decontamination program resulted in mitigation of a *C. auris* transmission cluster among our incarcerated patients. This investigation provides valuable insight into *C. auris* transmission in the incarcerated population, which is not considered a classic high-risk population as well as the challenges faced to stop transmission in a facility that requires the use of shared patient environments.

## Introduction

*Candida auris* is a yeast typically resistant to at least one antifungal drug, primarily fluconazole, with some cases resistant to all antifungal options. *C. auris* is also known to cause healthcare-associated infections and outbreaks. A unique characteristic is the difficulty of eradicating it from a physical environment once established even with effective agents.^1^ The U.S Environmental Protection Agency (EPA) List P details registered disinfectants with kill claims against *C. auris*. If one of these products cannot be selected, disinfectants from List K with a kill claim again *Clostridioides difficile* spores are acceptable alternatives.^2^ Communicable disease transmission is a particular challenge in prisons due to the nature of congregated living.^3,4^ Prior to summer 2022, there had been no individuals with *C. auris* identified in Texas prisons. There are currently no prevalence studies assessing the burden of this organism in this population. In fact, our literature review found no articles relating *C. auris* to a U.S. prison system. To our knowledge, this is the first described *C. auris* transmission cluster investigation and attempted mitigation strategy within any U.S. prison hospital system. This project was reviewed and approved by the Texas Department of Criminal Justice and the Institutional Review Board of the University of Texas Medical Branch.

## Methods

### Facility

The University of Texas Medical Branch-Texas Department of Criminal Justice (UTMB-TDCJ) Hospital Galveston (HG) is the first and only of its kind, maximum security hospital specializing in prisoners’ health care located on the campus of a major academic medical center. HG is comprised of approximately 50 patient rooms supporting 138 inpatient beds with a 26-bed telemetry unit and a 24-bed medical and surgical intensive care unit (ICU). Further, it houses an 82-bed hold over unit and an additional 85 open bay infirmary beds.

### Epidemiologic investigation

The index case was identified via a positive blood culture drawn on hospital day 15 from a febrile ICU patient admitted for altered mental status and respiratory failure. He had multiple comorbidities including diabetes mellitus, hemodialysis-dependent end-stage renal disease, hypertension, and a segmental pulmonary embolism. The patient was immediately placed in contact isolation, and his roommate was moved to a private room. Due the patient’s prolonged hospitalization, contact tracing focused on obtaining all prior room locations and roommates since admission. This was performed using the electronic medical record and clerical unit books maintained by staff for monitoring patient movement and cohorting. A list of exposed patients who had shared rooming with the index patient was compiled. The exposed patients along with their current roommates were screened using the CDC *Candida auris* surveillance recommendations.^5^ Using a Copan 480C eSwab, each patient’s bilateral axilla and groin locations were vigorously swabbed for culturing. Swabs were plated on chromogenic agar and incubated in the dark at 35° C ambient air for 72 hours. Alternatively, if chromogenic agar was not available, inhibitory mold agar incubated in the dark at 40° C ambient air was used. Plates were checked daily for visual growth. On days 2 and 3, a Wood’s lamp for fluorescence detection was utilized. Cultures were final at three days. Matrix-assisted laser desorption/ionization (MALDI) was performed for species identification with any indication of growth.

Through contact tracing, the ICU and an off-campus outpatient TDCJ infirmary unit, denoted Infirmary A, were identified as a commonality. Due to this, the ICU and Infirmary A were deemed high risk. We conducted intermittent ICU point prevalence screening surveys and had all patients screened who were admitted from Infirmary A to HG. Additionally, following discussions with Infirmary A’s leadership, it was recommended for patients housed in the off-campus Infirmary A’s main holding area undergo screening for *C. auris* as well.

### Environmental investigation

All rooms housing positive patients and their adjacent rooms were cleaned using EPA List P agents.^2^ A quaternary-ammonium-isopropyl alcohol-based germicidal wipe (Sani-Cloth®, PDI Healthcare, Woodcliff Lake, NJ) was used on medical equipment, and bleach disinfectant with 0.65% sodium hypochlorite (Dispatch®, Clorox Professional Products Co, Oakland, CA) was used for both daily and terminal cleaning of various high-touch surfaces by the environmental services team. Following terminal cleaning, environmental samples for culture were collected from various high-touch surfaces in the rooms, medical equipment, and shared staff workstations within HG. No environmental samples were collected from Infirmary A. High-touch surfaces included the door handles, bed rails, bedside chair (if present), bedside table, sink basin and handles, toilet seat, and thermostat. Medical equipment included intravenous pole(s), vitals monitor, and wall oxygen/suction ports. Staff workstations included a chair, counter space, telephone, keyboard, and mouse. Supplemental ultraviolet-C (UV-C) disinfection technology was utilized following terminal cleaning near the end of the investigation. Environmental samples were collected both pre- and post-UV-C irradiation to evaluate the efficacy of the UV-C device.

Environmental sampling for culture was performed using Copan 480C eSwabs. Surfaces were swabbed up and down their full length and included items within the space of the surface such as the call light and bed control panel. After sampling, each swab was deposited into 1 mL of liquid Amies transport media and taken immediately to the microbiology lab for culturing as previously described. In the event of a positive culture from a post-terminal clean environmental sample, the terminal cleaning and environmental sampling process was repeated. Positive rooms were closed until all environmental samples were culture negative.

### Whole genome sequencing and analysis

In collaboration with the Texas Department of State Health Services (DSHS), WGS was performed on the NextSeq 2000 platform (Illumina), with 300 cycles (150 bp PE) at the Utah Public Health Laboratory. WGS analysis was performed using the MycoSNP-nf bioinformatics pipeline for reference-based SNP calling. Comparator control samples were included in the analysis to help determine clade designation. The control samples were selected by the Mycotic Diseases Branch of the CDC and represent each major clade of *Candida auris*. A high-quality, complete reference genome from NCBI, GCA_016772135.1, was prepared for alignment and variant calling using the Burrows-Wheeler Aligner (BWA) nf-core module. The sequencing reads were prepared for Genome Analysis Toolkit (GATK) variant calling by quality assessment and trimming. Phred quality scores after trimming (≥28), GC content after trimming (42%–47.5%), and coverage (≥20X) were calculated, and it was found that all samples passed the quality metric thresholds set by the CDC. The alignment files were prepared using Picard and SAMTools nf-core modules. A multi-fasta consensus file was generated from GATK with only SNP positions and used as input into the Molecular Evolutionary Genetics Analysis (MEGA X) tool to construct a phylogenetic tree showing sequence similarity between each of the isolates. The branch lengths of the tree were customized to show SNPs and a Newick tree file (nwk) was exported to be used with Microreact for web-interface phylogenetic tree visualization benefits.

## Results

In total, 344 patients were screened during this investigation, 216 HG inpatients and 128 outpatient Infirmary A patients. All patients were male. The investigation identified nine patients positive for *C. auris* (Table 1). Our index patient was the only clinical case and the only death. The other eight patients were detected through surveillance screening for colonization. Eight cases were identified at HG, four of these patients presented from Infirmary A prior to admission. Of the 128 Infirmary A patients screened offsite, only one case (patient 4) was identified. In total, 33 primary contacts to the positive patients were identified. Of these, 20 underwent surveillance screening as the others had discharged, only one patient resulted positive deriving a secondary attack rate of 5%.

**Table 1.** Line list of all patients with positive *Candida auris* cultures including demographics, *C. auris* source, date of positive culture, and the patient’s contact, ICU, and outpatient infirmary histories

Patient	Sex	Age	Source	Test date	Contact status	Admitted to ICU prior to positive testing	Infirmary unit prior to admission
1	M	60	Blood	6/28/2022	Index Case	No	A
2	M	54	Axilla/Groin Swab	7/4/2022	Secondary (Patient 3)	Yes	A
3	M	66	Axilla/Groin Swab	7/7/2022	Primary (Index)	Yes	B
4	M	56	Axilla/Groin Swab	7/15/2022	Infirmary A PPS	No	A
5	M	38	Axilla/Groin Swab	7/18/2022	ICU PPS	Yes	C
6	M	25	Axilla/Groin Swab	7/18/2022	ICU PPS	Yes	County Jail
7	M	65	Axilla/Groin Swab	7/25/2022	Admission	Yes	A
8	M	65	Axilla/Groin Swab	7/25/2022	ICU PPS	Yes	D
9	M	54	Axilla/Groin Swab	7/29/2022	Admission	No	A

Note. ICU: intensive care unit; PPS: point prevalence survey.

We identified two patients with positive surveillance screens from the index patient’s initial contact tracing which included both primary and secondary contacts. Additional contact tracing began for the newly identified patients as previously outlined. Outside of Infirmary A, we found that HG’s ICU population was disproportionally affected, thus universal isolation was implemented for all patient care activities on this unit. We conducted intermittent point prevalence surveys of all ICU patients. Once we had a negative point prevalence survey and no new cases were identified through contact tracing, universal isolation in the ICU and additional point prevalence surveys were discontinued.

WGS analysis of the nine patient isolates showed that all isolates were clade III and closely clustered together. The largest SNP difference between the source patient isolate and others was 21 SNPs. This lends evidence that the transmission cluster stemmed from a single common source.

In total, 527 environmental samples were collected during the investigation. Sixty-one rooms underwent post-terminal cleaning environmental sampling, and seven rooms (11.5%) had positive cultures with 15 high-touch sites resulting culture positive between them (Table 2). Of the UV-C focused samples, 27 pre-UV-C samples and 26 post-UV-C samples were obtained. Two sites from one room, bed/bedside chair and bedside table, resulted positive both pre- and post-UV-C treatment. Repeat terminal cleaning and UV-C disinfection were performed in this room with negative follow-up environmental cultures.

**Table 2.** Environmental sample sources with the number of positive cultures following terminal disinfection

Environmental source	Positive cultures
Hospital Bed Rails/Bedside Chair	5 (1*)
Bedside Table	4 (1*)
Door	2
Toilet Seat	2
IV Pole/Blood Pressure Machine	1
Unknown Site	1
Sink Basin/Handles	0
Wall Oxygen and Suction Ports	0
Vitals Monitor	0
Staff Workstation	0
Thermostat	0

*Sites positive following UV-C irradiation.

## Discussion

Our transmission cluster investigation highlights the continued spread of *C. auris* among vulnerable populations. Until recently, *C. auris* was mainly a concern among post-acute care facilities such as long-term care hospitals (LTACH) and ventilator-capable skilled-nursing facilities (vSNF). This risk distribution is driven by a patient population with more comorbid conditions and complex medical care needs requiring prolonged healthcare exposure. Additionally, effective infection control practices have been difficult to maintain at these facilities.^6,7^ This was evident by a large outbreak of *C. auris* encompassing 17 post-acute care facilities where lapses in isolation precautions, environmental cleaning, and hand hygiene were noted to contribute to the outbreak’s propagation.^8^ However, a recent CDC survey of reported *C. auris* clinical cases and colonization rates from various state and local health departments across the United States and the CDC’s Antimicrobial Resistance Laboratory Network noted a 95% and 200% increase, respectively, from 2019 to 2021. A significant part of this was attributed to increases in screening efforts. Further, this report highlighted the growing geographic spread of *C. auris* with an increase in new transmission across Texas.^6,9^

What is not found in these reports of *C. auris* transmission is the impact faced by incarcerated populations. At first glance, this is not a population classically defined as high-risk. Per CDC guidance, the highest risk are those with exposure to nursing facilities with indwelling devices and/or lines such as endotracheal or tracheostomy tubes, feeding tubes, and central lines.^10^ While these risks are present in our infirmed incarcerated population, one must also speculate the effect congregate settings has on additional spread. Outpatient infirmary units are not closed units; thus, these inmates can be released back to the prison’s general population and as can be expected, released from prison back to the community. Communicable disease transmission in incarcerated populations has been well documented historically with tuberculosis and most recently with COVID-19.^11,12^ However, unlike tuberculosis and COVID-19 which are spread via droplet and droplet nuclei, *C. auris* is predominately transmitted via direct person-to-person contact and/or indirect contact with contaminated fomites in the environment.^2,6,10^ A similar surrogate organism would be methicillin-resistant *Staphylococcus aureus* (MRSA), which is a known problem among the incarcerated, most similarly, among TDCJ inmates.^13^ In a recent prevalence study evaluating acquisition of new MRSA colonization on day 30 of incarceration, an 8.4% acquisition rate was detected. While there are several ways for incarcerated persons to acquire MRSA, shared housing, particularly dorm and cell-based living units, had more overlap than would be expected by chance alone.^14^ Further, modeling has shown that as the incarcerated population increases, so do MRSA colonization rates and thus the potential for increased community spread.^14,15^ This is of particular interest as the US has the highest incarceration rate in the world. Additionally, Texas is the leading contributor to both state and federal prison populations and for prison releases back to the community.^16^

Our cluster was caused by the clade III (Africa) strain of *C. auris*. Geographically, this is a common strain in our area.^17,18^ Despite the geographical commonality between the community and hospital clades, we do not believe this reflects community-based acquisition of these cases prior to incarceration. Our hospitalized case cluster occurred in a defined space and time, and the transmission was abated with infection control practices. This suggests an internal transmission cluster. Regarding disinfection, clade III strains have been shown to be more difficult to eradicate.^19,20^ While the EPA lists several agents with kill claims against *C. auris*, we elected to primarily use bleach in our cleaning processes. This is largely because bleach is readily available and affordable. Additionally, sporicidal agents have demonstrated better log reductions compared with quaternary-ammonia based products.^21^ We used a quaternary-ammonia-isopropyl alcohol product on medical equipment where bleach would have been too corrosive. Data have supported the use of quaternary-ammonia-isopropyl alcohol agents against *C. auris*.^21^

Bleach-based cleaning was fairly effective in the decontamination process; however, the 11.5% post-terminal cleaning environmental culture positivity rate noted in our investigation can still pose a problem in maintaining transmission. We, therefore, conducted a preliminary assessment of supplemental UV-C irradiation in our terminal cleaning process, where one room had positive post-UV-C cultures. The room underwent repeat terminal cleaning and UV-C disinfection, and follow-up cultures were negative. Given this, no reliable conclusions can be drawn regarding UV-C efficacy from this investigation. Regarding UV-C disinfection against *C. auris* clades, it should be noted that clade III was found to have the least log-kill even with extended UV exposure times compared to clades I, II, and IV. This is believed to be due to the proclivity of clade III to form aggregates which may make UV penetration more difficult.^20,22^ Since this investigation, we have trialed other UV-C devices in the same manner and have found that exposure time adjustments have been necessary to achieve culture negativity (data not presented).

While a *C. auris* admission surveillance screening policy was in place for our non-prison hospital system for patients presenting from outside nursing facilities, it was not implemented within HG. The reasons for this were several, the largest being that prison units were not considered high risk for *C. auris*. Additionally, while HG is attached to our academic medical center and we share medical staff and some administrative departments, it is an independent healthcare institution with its own leadership including a warden. Further, we faced many logistical challenges such as limited overall available inpatient rooms with a need for shared rooming, shared medical equipment, a need for non-healthcare trained TDCJ personnel to interface with patients, and frequent patient movements/relocations that made policy implementation challenging. However, during this investigation, admission screening for all patients from Infirmary A was implemented. We have since expanded to universal screening for all admissions to HG regardless of their outpatient location (infirmary or general population). Additionally, we collaborated with Texas DSHS and TDCJ Correctional Managed Care to develop a *C. auris* infection control policy for infected and colonized inmates throughout the Texas prison system that non-healthcare trained TDCJ personnel can follow.

Another challenge faced was the use of a culture-based surveillance process that required contact isolation to be followed until the surveillance screening cultures finalized negative at three days. Thus, this limited cohorting screen-pending patients with others. We have since switched to a PCR-based surveillance screen rather than culture-based. This has dramatically reduced the turnaround time for results and thus has lowered overall patient isolation times and PPE usage. While a direct source for this transmission cluster was not clearly identified, we speculate that shared equipment/environments likely contributed. These patients are in almost constant contact with one another even while admitted to HG. At times, up to three patients are housed in one room together without partitions between beds. As expected, areas with the most patient contact were more likely to be culture positive despite terminal cleaning indicating a higher bioburden in those areas. A commonality among several of the patients was Infirmary A. As stated, this infirmary performed its own patient surveillance screening; however, only one patient was found from that effort and no environmental samples were collected.

In summary, this report provides valuable insight into *C. auris* transmission in the incarcerated hospitalized population which is not considered a classic high-risk population. However, high-risk infirmed patients are prevalent throughout the prison system. Additionally, those who are released back into the community may find themselves in residential reentry centers with at-risk individuals or living with at-risk family or friends for which *C. auris* infection could carry significant morbidity and mortality. Given our findings, we have continued bleach-based daily and terminal cleanings and UV-C disinfection for all *C. auris* isolation rooms. Additionally, we expanded our *C. auris* admission screening process to all inmate admissions as well as collaborated with Texas DSHS and TDCJ Correctional Managed Care to develop a *C. auris* infection control policy for the infected and colonized inmates of the Texas prison system at large. Further work is needed to assess the impact incarcerated populations have on *C. auris* transmission both within the incarcerated and general community populations.
